# Clinical Features and Computed Tomography Findings Are Utilized to Characterize Retrobulbar Disease in Dogs

**DOI:** 10.3389/fvets.2018.00186

**Published:** 2018-08-21

**Authors:** Jenna N. Winer, Frank J. M. Verstraete, Derek D. Cissell, Catherine Le, Natalia Vapniarsky, Kathryn L. Good, Claudio J. Gutierrez, Boaz Arzi

**Affiliations:** ^1^School of Veterinary Medicine, William R. Pritchard Veterinary Medical Teaching Hospital, University of California, Davis, Davis, CA, United States; ^2^Department of Surgical and Radiological Sciences, University of California, Davis, Davis, CA, United States; ^3^Department of Pathology, Microbiology and Immunology, School of Veterinary Medicine, University of California, Davis, Davis, CA, United States; ^4^Department of Anatomy, Physiology and Cell Biology, School of Veterinary Medicine, University of California, Davis, Davis, CA, United States

**Keywords:** canine, orbit, exophthalmos, neoplasia, infection, inflammation, retropulsion

## Abstract

The objective of this study is to describe the clinical features and computed tomography (CT) findings of dogs with retrobulbar disease. There are two facets to this study: a retrospective case series in which findings of dogs with primary vs. secondary retrobulbar disease are described, and a retrospective cross-sectional study in which computed tomography findings of dogs with retrobulbar neoplasia vs. infection/inflammation are described and compared. The medical records of 66 client-owned dogs diagnosed with retrobulbar disease between 2006 and 2016 were reviewed. Clinical information including signalment, the specialty service to which the dog was presented, clinical signs, physical examination findings, diagnostic results, treatment, and outcome were documented. Diagnostic imaging and histopathology were reviewed. Forty-one dogs (62.1%) were diagnosed with primary disease of the retrobulbar space; 25 dogs (37.9%) were considered to have secondary retrobulbar disease. Of the 41 dogs with primary retrobulbar disease, 19 were diagnosed with neoplasia, 19 with infectious/inflammatory disease, and 3 suffered traumatic insult to the retrobulbar space. Of the 25 dogs with secondary retrobulbar disease, 21 were diagnosed with neoplasia, 3 with infectious/inflammatory disease, and 1 with a cyst. Dogs had a combination of ocular, oral, and/or nasal clinical signs. CT findings of orbital osteolysis, orbital periosteal reaction, and presence of a retrobulbar mass were significantly associated with neoplasia, while zygomatic salivary gland enlargement, retrobulbar mass effect, and mandibular lymphadenopathy were more often associated with infectious/inflammatory disease. CT findings overlap among different retrobulbar diseases, but new bone formation and lysis are more often associated with neoplasia. Disease originating from the retrobulbar space was equally likely to be infectious/inflammatory (*n* = 19) or neoplastic (*n* = 19), based on definitive diagnostic results of dogs with primary retrobulbar disease. Due to the clinical ramifications of these disorders, the diagnosis and treatment of these cases should be managed with a multi-specialty approach.

## Introduction

**Figure 1:**
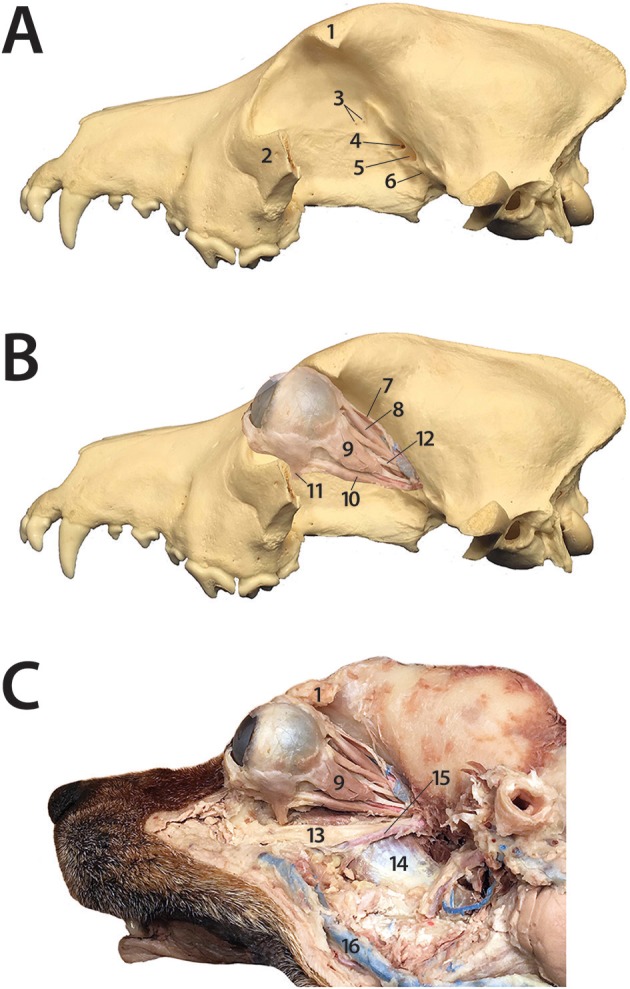
Dog prosection demonstrating the complex anatomy of the orbit and retrobulbar space. **(A)** 1: Zygomatic process of the frontal bone; 2: Part of the zygomatic arch (the rest of which has been removed); 3: Ethmoid foramina; 4: Optic canal; 5: Orbital fissure; 6: Rostral alar foramen **(B)** 7: Upper eyelid levator muscle; 8: Dorsal rectus muscle; 9: Lateral rectus muscle; 10: Ventral rectus muscle; 11: Ventral oblique muscle; 12: Oculomotor nerve **(C)** 1: Zygomatic process of the frontal bone; 13: Maxillary nerve; 14: Medial pterygoid muscle; 15: Maxillary artery; 9: Lateral rectus muscle; 16: Facial vein.

Retrobulbar disorders are challenging to diagnose and treat, due to the array of possible clinical presentations and anatomic complexity of the region. The retrobulbar space is the area within the orbit that lies caudal to the globe. Six bones contribute to the structure of the orbit: frontal, lacrimal, maxillary, zygomatic, palatine, and sphenoid (1, 2). The orbit of dogs is not a complete bony structure. The lateral wall is bound by both the lateral orbital ligament and medial surface of the temporal muscle, while the ventral floor is formed by soft tissues that include the medial pterygoid muscle, zygomatic salivary gland, and orbital adipose tissue (1, 3). The retrobulbar tissues of the dog include extraocular muscles, the lacrimal gland, the zygomatic salivary gland, masticatory muscles, nerves, blood vessels, and the bones that form the orbit (Figure 1). The orbit contains a cone of eye-associated structures termed the periorbita, the apex of which points caudally. The periorbita is derived from the periosteum, encloses the caudal portion of the eyeball and the extrinsic muscles of the eye, and is surrounded by orbital fat. The extrinsic muscles of the eye that are contained within and visible through the periorbita are the dorsal, ventral, lateral and medial rectus muscles, the retractor bulbi with its four fascicles, and more superficially and medially, the dorsal oblique muscle. All of the extrinsic muscles of the eye except for the ventral oblique originate in the apex of the orbit (1, 4–6). The orbit serves to position and protect the globe and house the retrobulbar tissues (2).

The orbit, and thus the retrobulbar contents it contains, is intimately related to many other structures of the head. It abuts the nasal cavity and paranasal sinuses, which are located medial and dorsal to the orbit; it is closely associated with the cranial cavity, which is located caudomedial to the orbit; and it is adjacent to the mouth, which is located ventral to the orbit (7). The apices of the maxillary fourth premolar tooth and first and second molar teeth are in close proximity to the orbital floor, separated from ventral orbital soft tissues by a thin layer of alveolar bone (8). The ramus and condylar process of the mandible lie caudal and lateral to the retrobulbar space (1).

The anatomical relationship of the orbit to nearby structures, and its diverse variety of retrobulbar contents, renders this area vulnerable to a wide variety of pathology. Diseases may arise primarily from or within the retrobulbar tissues and remain confined to the orbit or expand outwards. Conversely, diseases of adjacent structures may extend into the retrobulbar space, referred to as secondary retrobulbar disorders (5, 7). Primary retrobulbar pathology includes neoplasia (9), retrobulbar cellulitis or abscessation (e.g., idiopathic or secondary to a foreign body), zygomatic salivary gland mucocele or sialoadenitis, congenital and developmental disorders (e.g., cysts or arteriovenous fistulae) (10), masticatory muscle myositis (11) and extraocular polymyositis (12), hematoma (13), and direct trauma (5). Secondary retrobulbar pathology includes ingrowth of neoplasia from an adjacent site (e.g., the nasal cavity) or metastasis from a distant site (14–17) and spread of nearby infection or inflammation [e.g., extension of endodontal or periodontal-endodontal dental lesions (18, 19), severe rhinitis or sinusitis (20)].

Because the orbit is a semiclosed space, the pathognomonic sign of a space-occupying retrobulbar lesion, although not always present, is exophthalmos (5, 21, 22). Additional clinical signs that can be seen with retrobulbar disease include decreased ocular retropulsion, strabismus, protrusion of the nictitating membrane, pain on periorbital palpation, and blindness (5, 6, 10). If an orbital lesion compresses the orbital veins, venous drainage becomes diminished and thus chemosis and increased intraocular pressure can occur (21). Importantly, when the oral cavity is opened, the ramus of the mandible rotates rostrally, exerting pressure on the orbital contents, eliciting symptoms of pain (21).

Because clinical signs can be nebulous, and direct examination of the orbit is limited, additional diagnostic modalities are often necessary to determine the specific diagnosis, and thus treatment and prognosis (7, 14, 23–25). Cross-sectional imaging modalities, namely ultrasonography, computed tomography (CT), and magnetic resonance imaging (MRI), enable detailed evaluation of the orbit and can allow clinicians to characterize, localize, and determine the extent of retrobulbar disease (14, 26, 27). Imaging alone may be sufficient to determine the biologic behavior of the disorder and thus hint at its underlying etiology (14, 28–30); however, further testing is necessary to achieve a definitive diagnosis, such as cytological, histopathological, or microbiological examination of orbital samples (20).

Disorders of the retrobulbar space are varied and complex; hence, diagnosis and management of these disorders may not be straightforward. The purpose of the present study is to describe the clinical and CT features of a large cohort of dogs with primary and secondary retrobulbar disorders, and to review treatments pursued and treatment outcomes. Regarding CT features, we hypothesize that specific imaging findings would be more commonly associated with different underlying disease processes (e.g., infectious/inflammatory or neoplastic).

## Materials and methods

### Case selection criteria

The electronic medical records database of the University of California-Davis William R. Pritchard Veterinary Medical Teaching Hospital (UCD-VMTH) was searched to identify dogs examined between January 2006 and December 2016 (11 years inclusive) that were diagnosed with retrobulbar disease. Therefore, this study is a retrospective case series. The main basis for inclusion in the study was pathology involving the retrobulbar space evident by CT; CT reports were searched for the keyword “retrobulbar.” Cases were included if an etiological diagnosis was achieved by cytology, biopsy, bacterial or fungal culture, exploratory surgery, necropsy and/or positive response to antibiotics or anti-inflammatory medications. Cases were excluded if diagnostic results were inconclusive.

### Medical records review

Medical records were reviewed to obtain the following information: age at time of diagnosis; sex; breed; the UCD-VMTH specialty service to which the dog was originally presented; clinical signs described by the owner; physical examination findings at the visit during which retrobulbar disease was discovered; clinicopathologic or histopathologic results derived from the retrobulbar space and the diagnostic modality utilized (e.g., retrobulbar biopsy, fine needle aspiration and cytology, bacterial culture and sensitivity, or fungal culture); diagnosis to the highest level of understanding; treatment(s) pursued; and the outcome, if known. Cases were categorized as primary vs. secondary retrobulbar disease, and further categorized based on diagnosis as either infectious/inflammatory, neoplastic, traumatic, or other.

Clinical signs, as reported by the owner, were reviewed for each dog. Clinical signs were divided into the following categories: ocular signs (e.g., exophthalmos, periorbital swelling, conjunctival and/or episcleral hyperemia, chemosis, blepharospasm, and/or ocular discharge); oral signs (e.g., pain when chewing or reluctance to open the mouth); and nasal signs (e.g., epistaxis, nasal discharge, sneezing, and/or stertorous breathing). Dogs were recorded as fitting into the above 3 categories, or if multiple different clinical signs were reported, were placed into a combination category (e.g., ocular signs and oral signs).

Physical examination findings potentially relating to retrobulbar pathology were grouped similarly to clinical signs—namely, if ocular findings, oral findings, or nasal findings were appreciated by the clinician. Ocular findings, as considered for this study, included decreased ocular retropulsion, strabismus and/or exophthalmos. Oral findings included apparent pain or discomfort on opening the mouth, decreased range of motion of the temporomandibular joint, and/or a mass or hemorrhagic discharge visible within the oral cavity. Nasal findings included decreased airflow, epistaxis, serosanguinous and/or mucoid nasal discharge.

Treatment was broadly divided into medical or surgical management. Medical management was categorized as follows: antimicrobial therapy +/– anti-inflammatory (prescribed to achieve a definitive cure) or radiation therapy/chemotherapy (pursued with either palliative or curative intent). If a course of analgesic, antibiotic, or anti-inflammatory medications were prescribed with the intention of providing palliation and not definitive cure, this was not registered as medical management. Surgical management was divided into the following categories: enucleation or exenteration, mass excision, surgical exploration with abscess drainage, or dental extractions.

As part of the initial medical records review, CT reports were initially screened in order to divide dogs into 2 groups: those with retrobulbar disease considered to be primary vs. those with retrobulbar disease considered to be secondary. The entire CT images were than randomly reviewed by a board-certified radiologist (see next section). This allocation was used to analyze cases as it follows the authors' clinical approach to dogs with retrobulbar disorders: first determine if the disease is primary or secondary, and then determine underlying etiology. Dogs were divided into subgroup (infectious/inflammatory disease vs. neoplasia) based on diagnosis according to results of cytology, biopsy, bacterial or fungal culture, exploratory surgery, necropsy and/or positive response to antibiotics or anti-inflammatory medications.

### Radiologic examination

CT images were acquired with either a single-slice or 16-slice helical scanner (HiSpeed FX/i or LightSpeed16, GE Healthcare, Waukesha, WI). CT images were reviewed by 2 authors (JNW and DDC, a board-certified radiologist) in transverse and dorsal planes using a bone window (WW = 2,900, WL = 600) and soft tissue window (WW = 350, WL = 40). CT images were reviewed in a randomized order, and reviewers were blinded to the diagnosis (i.e., whether the lesion was infectious/inflammatory, neoplastic, or other). CT reports that had been read during the initial medical records review were no longer referenced and instead new observations and conclusions were made by directly viewing CT images by authors JNW and DDC. Post-contrast images, acquired after intravenous administration of iopamidol (Isovue 370, Bracco Diagnostics, Monroe Township, NJ), were reviewed when available and compared to pre-contrast images. The orbital walls and zygomatic bone were evaluated for evidence of distortion, osteolysis, or periosteal reaction (scored as present or absent). The retrobulbar space was evaluated for reduced presence of fat (scored subjectively as the same amount of fat or as less fat than the contralateral, unaffected retrobulbar space). Osseous distortion of the orbit and reduction of retrobulbar fat were assessed by Fisher's exact test. A Bonferroni corrected *p* ≤ 0.0042 was used to define statistical significance. The retrobulbar space was further evaluated for the presence of a mass effect or for the presence of a mass (scored as present or absent). A mass effect was defined as displacement or distortion of retrobulbar structures in the absence of a definable mass. When a mass was present, its Hounsfield units (HU) were measured on both pre-contrast and post-contrast images, and the pattern of contrast enhancement was noted (no enhancement, peripheral enhancement, heterogeneous enhancement, or homogenous enhancement). The zygomatic salivary gland was evaluated for enlargement, compression, or displacement (scored subjectively compared to the contralateral, unaffected retrobulbar space). The globes were evaluated for exophthalmos, enophthalmos, buphthalmos, or deformation (scored subjectively compared to the contralateral, unaffected retrobulbar space). The mandibular, medial retropharyngeal, and superficial cervical lymph nodes (when included in the scan) were evaluated for evidence of enlargement (scored subjectively compared to the contralateral, unaffected side).

### Histologic examination

All available histopathology slides were reviewed by a board-certified pathologist (NV). Representative photomicrographs of each retrobulbar disease category (infectious/inflammatory and neoplastic retrobulbar disease) are included within this manuscript.

### Statistical analysis

There are two facets to this study: a retrospective case series in which findings of dogs with primary vs. secondary retrobulbar disease are described, and a retrospective cross-sectional study in which computed tomography findings of dogs with retrobulbar neoplasia vs. infection/inflammation are described and compared. Therefore, data was analyzed in two fashions.

First, the entire sample of 66 dogs was divided into two groups (i.e., primary and secondary retrobulbar disease) based on a review of the medical records, and then further subdivided by disease etiology based on definitive diagnostic testing. For this portion of the study, statistical analysis comprised summary statistics for demographics, clinical signs, physical exam findings, treatment, and outcome.

Second, the sample population was again divided into two groups (i.e., infectious/inflammatory vs. neoplastic disease) based on definitive diagnostic testing, and then the CT findings of patients with each etiology were compared. Potential differences between infectious/inflammatory and neoplastic retrobulbar disease were assessed by Pearson's Chi-square test for independence for each CT finding for which the expected counts of each cell of its 2 × 2 contingency table was ≥5. Two-tailed Fisher's exact test was used to assess CT findings that were present in ≥20% of dogs in at least one disease category, but did not meet the criteria for Pearson's Chi-square test. Statistical significance was defined for CT findings by *p* ≤ 0.05 with a Bonferroni correction for the total number of Chi-square and Fisher's exact tests performed. Sensitivity and specificity for diagnosis of neoplasia or infectious/inflammatory disease were calculated for CT findings with a statistically significant relationship to category of disease. All patients were included in calculation of sensitivity and specificity, as all categories of disease may contribute false positive or false negative observations. Pre-contrast HU and percent increase in HU following contrast administration were compared between inflammatory and neoplastic masses by Mann-Whitney U test. Differences in patterns of contrast enhancement between inflammatory and neoplastic masses were compared by Fisher's exact test.

## Results

Between January 2006 and December 2016, 575 dogs underwent skull CT at our institution; out of those, 84 dogs underwent CT examination for which a CT report was generated that contained the word “retrobulbar.” Upon further review of the CT reports, 4 dogs were excluded from this study because the report discussed the retrobulbar space in normal anatomical terms. An additional 14 dogs were excluded because a clear diagnosis was not reached, either because diagnostic testing beyond the CT scan was not performed, or because diagnostic tests that were performed provided contradictory or inconclusive results. Thus, data from 66 dogs are presented in this study. Of the 66 dogs, 41 (62.1%) were deemed to have primary disease arising from the retrobulbar space. The other 25 dogs (37.9%) were diagnosed with secondary retrobulbar disease.

### Primary retrobulbar disease

**Figure 2:**
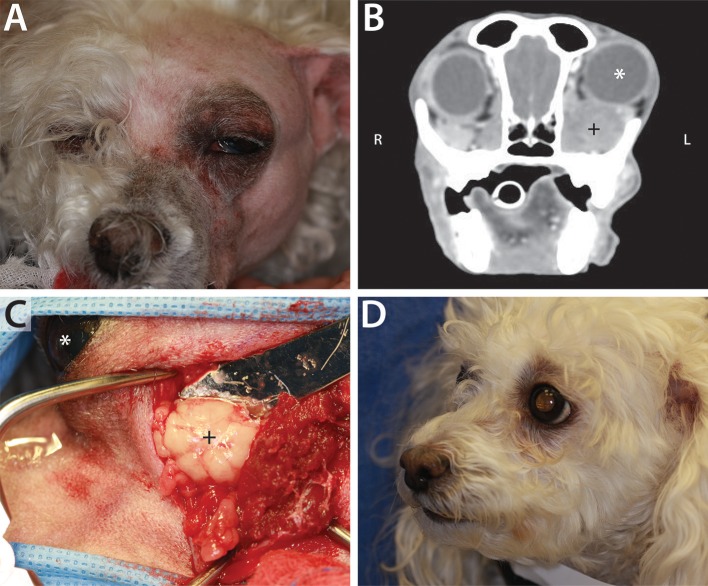
Example of neoplastic primary retrobulbar disease in a 10-year-old female spayed toy poodle. **(A)** Clinical appearance of the dog just prior to surgical intervention. Note the left exophthalmos and periocular swelling. **(B)** Post intravenous contrast administration transverse CT image of this dog, demonstrating left exophthalmos (white ^*^) secondary to a heterogeneously contrast enhancing mass (+). **(C)** Intraoperative photograph during orbitectomy, zygomatectomy, and tumor excision, showing the mass (+). The left eye (white ^*^) is included in the surgical field for orientation, with the patient positioned in right lateral recumbency and rostral pointing to the left of the photograph. Histopathology confirmed a diagnosis of retrobulbar liposarcoma. **(D)** Clinical appearance of patient at 4-months recheck post retrobulbar liposarcoma excision. There was no clinical evidence of tumor recurrence 7-months postoperatively, at which point the dog was lost to follow up.

**Figure 3:**
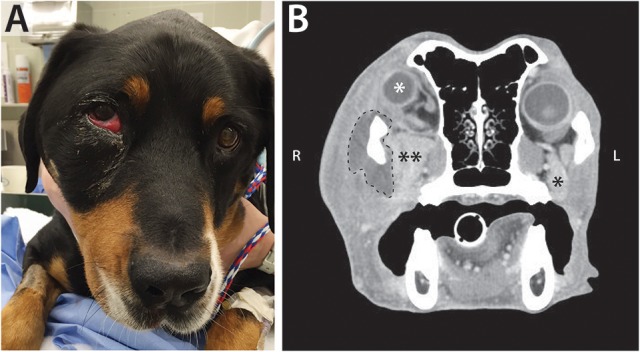
**(A)** Clinical appearance of a 6-year-old male castrated Swiss mountain dog that was presented to the Ophthalmology Service for ocular (right exophthalmos with periorbital swelling) and oral (ptyalism, vocalizing when eating) clinical signs. **(B)** CT scan contributed to a diagnosis of retrobulbar abscess secondary to plant foreign material. Post intravenous contrast administration transverse CT image shows right exophthalmos (white *), enlargement of the right zygomatic salivary gland (^**^) compared to the left (^*^), and a mass effect surrounding the right zygoma (dashed line). Surgical exploration and drainage of the abscess, followed by medical management, allowed for resolution of the primary retrobulbar infection.

Of the 41 dogs with primary retrobulbar disease, 19 (46.3%) were diagnosed with neoplasia (Figure 2), 19 (46.3%) were diagnosed with infectious/inflammatory disease (Figure 3), and 3 (7.3%) suffered traumatic insult to the retrobulbar space (i.e., unknown blunt trauma, dog bite wound, and vehicular accident).

Age of the dogs with primary retrobulbar disease ranged from 1.5 months to 14 years, with a mean age of 7.5 years. Dogs with primary retrobulbar neoplasia ranged in age from 7 months to 12 years (mean 8.5 years, median 10 years). Dogs with primary infectious/inflammatory retrobulbar disease ranged in age from 1 year to 13 years (mean 7.0 years, median 6 years). The 3 dogs that suffered from retrobulbar trauma were aged 1.5 months, 2 years, and 13 years.

There was 1 intact male, 8 castrated males, and 10 spayed females with neoplastic primary retrobulbar disease. There were 5 intact males, 7 castrated males, and 7 spayed females with infectious/inflammatory primary retrobulbar disease.

Breeds of the 19 dogs with primary retrobulbar neoplasia included 3 boxers, 2 Brittany's, and 2 terrier mixes. The 19 dogs with primary infectious/inflammatory retrobulbar disease included 3 Labrador retriever/Labrador mixes and 2 Yorkshire terriers. The remaining 26 dogs with primary retrobulbar disease represented 26 other purebred or mixed breeds.

Specialty services to which dogs with retrobulbar disease were initially presented are listed in Table 1. Clinical signs as reported by owners compared to specialty service to which dogs were initially presented are listed in Table 2.

**Table 1:** The number of dogs with primary (P) and secondary (S) retrobulbar pathology that were presented to each specialty service.

	**Infectious/Inflammatory**		**Neoplasia**		**Trauma**		**Other**	
	**P**	**S**	**P**	**S**	**P**	**S**	**P**	**S**
Ophthalmology	8	0	14	3	0	0	0	0
Emergency	9	0	0	1	0	0	0	0
Dentistry and oral surgery	1	2	1	0	1	0	0	0
Internal medicine	0	1	1	7	0	0	0	0
Neurology	1	0	0	0	2	0	0	0
Medical oncology	0	0	2	5	0	0	0	0
Radiation oncology	0	0	1	5	0	0	0	0
Soft Tissue surgery	0	0	0	0	0	0	0	1

*The rows are organized by specialty service to which the dog was originally presented; the columns are organized by disease etiology*.

**Table 2:** Clinical signs as reported by owners in dogs that were diagnosed with primary retrobulbar disease as compared to the specialty service to which the dog was initially presented.

	**Ocular signs**	**Oral signs**	**Nasal signs**	**Ocular + Oral**	**Ocular + Nasal**	**Oral + Nasal**	**No clinical signs associated with retrobulbar disease**
Ophthalmology	18			3	1		
Emergency	2	2		4		1	
Dentistry and oral surgery	1	1		1			
Neurology	2	1					
Internal medicine	1						
Medical oncology	1						1
Radiation oncology	1						

“*Ocular signs” include exophthalmos, periorbital swelling, blepharospasm, ocular discharge, or eye redness. “Oral signs” include pain when chewing, reluctance to open mouth. “Nasal signs” include epistaxis, nasal discharge, sneezing, stertorous breathing. If multiple different clinical signs were reported, a combination category was notated (i.e., ocular signs + oral signs)*.

**Figure 4:**
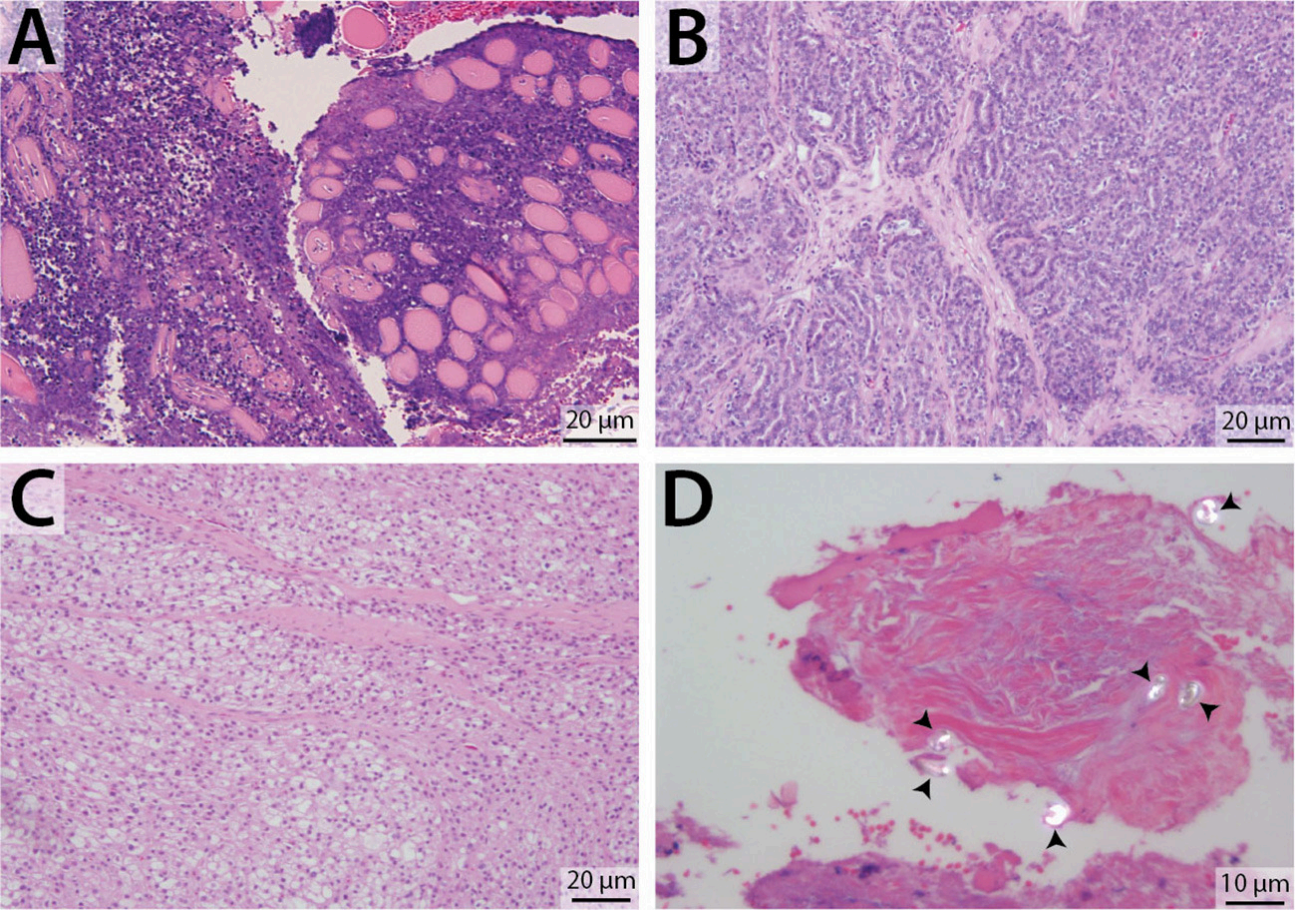
Representative photomicrographs of retrobulbar biopsy specimens. H&E stain, with scale bars included within images. **(A)** Degenerate and necrotic myocytes separated by mixed inflammatory cellular infiltrate. **(B)** Zygomatic salivary adenocarcinoma. **(C)** Liposarcoma (biopsy from the dog featured in Figure 2). **(D)** Polarized light demonstrates the presence of multiple birefringent foreign substance, consistent with plant material (arrowheads) (biopsy from the dog featured in Figure 3).

Biopsy with histopathology of tissue from the retrobulbar space was performed in 19 dogs (Figure 4), fine needle aspiration with cytology was performed in 23 dogs, bacterial culture and sensitivity was performed in 20 dogs, and fungal culture was performed in 5 dogs. Histopathology was considered definitive for 17/19 (89.5%) dogs; cytologic diagnosis was considered definitive for 18/23 (78.2%) dogs. Of the dogs that underwent both histological and cytological testing (*n* = 14), 9 (64.3%) had complimentary conclusive test results, 3 (21.4%) had definitive histopathology results but inconclusive cytology results, 1 (7.1%) had definitive cytology results but inconclusive histopathology results, and 1 (7.1%) had both inconclusive histopathology and cytology results. Of the 19 dogs diagnosed with primary infectious/inflammatory retrobulbar disease, 14 underwent bacterial +/– fungal culture, and 8 (57.1%) received culture results consistent with infection (i.e., microbial growth). None of the dogs included in this study was diagnosed with masticatory muscle myositis or extraocular polymyositis.

All 19 dogs diagnosed with primary infectious/inflammatory retrobulbar disease were prescribed various antimicrobial and anti-inflammatory medications. Surgical management was additionally pursued in 7 dogs−5 dogs underwent globe sparing surgical exploration with abscess debridement and 2 underwent enucleation or exenteration. Treatment outcome could not be determined for 7 dogs that were lost to follow-up. Twelve dogs either positively responded to medical management or achieved clinical resolution of their retrobulbar disease.

Of the 19 dogs with primary neoplastic retrobulbar disease, 1 was managed medically with radiation therapy/chemotherapy, 5 were managed surgically [3 underwent enucleation or exenteration, 2 underwent tumor excision (Figure 2)], and 3 were managed both surgically and medically (enucleation or exenteration followed by radiation therapy and/or chemotherapy). Treatment was not pursued for 7 dogs and they were lost to follow-up. Euthanasia was performed on 3 dogs in lieu of pursuing treatment.

### Secondary retrobulbar disease

**Figure 5:**
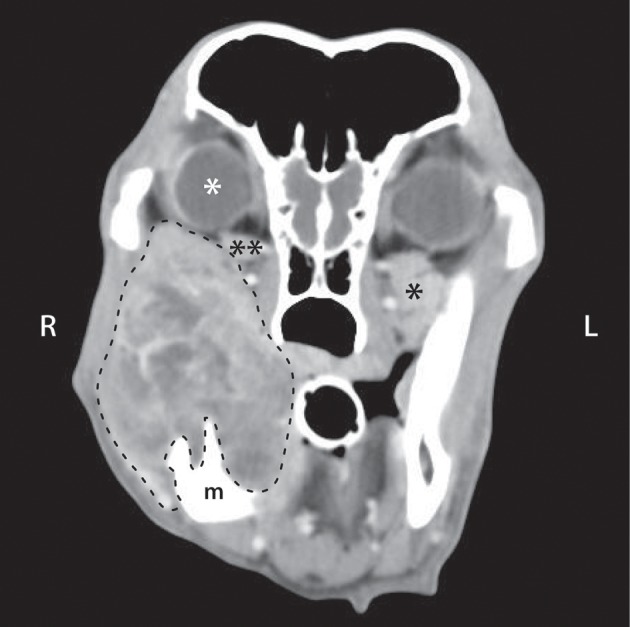
Post intravenous contrast administration transverse CT image of a 10-year-old male castrated golden retriever that was diagnosed with a right caudal mandibular malignant melanoma. CT scan shows a heterogeneously contrast enhancing mass arising from the right mandible (m), with secondary retrobulbar extension of the mass (dashed line). There is right ocular exophthalmos (white ^*^), compression of the right zygomatic salivary gland (^**^) compared to the left (^*^), and a decrease in retrobulbar fat (darkest gray color in the retrobulbar space).

Of the 25 dogs with secondary retrobulbar disease, 21 (84.0%) were diagnosed with neoplasia arising from an adjacent anatomical structure with extension into the retrobulbar space (Figure 5). The other 4 dogs had the following diagnoses: a maxillary odontogenic cyst with retrobulbar extension, retrobulbar cellulitis with abscessation secondary to maxillary first and second molar tooth periapical infection, and 2 dogs with a sterile retrobulbar abscess presumed secondary to nasal cavity neoplasia.

Age of the dogs at which their secondary retrobulbar disease was first discovered at the VMTH ranged from 4 to 14 years, with a mean age of 10.2 years (median 10 years). Sex distribution of the 30 dogs with secondary retrobulbar disease was as follows: 1 intact male, 11 castrated males, 1 intact female, and 12 spayed females.

Of the 21 dogs with secondary retrobulbar neoplasia, 5 were Labrador retriever/Labrador mix dogs and 3 were purebred or mixed golden retrievers. The remaining cases of secondary retrobulbar disease included 17 other purebred or mixed breeds.

Specialty services to which dogs with primary and secondary retrobulbar disease were initially presented is summarized in Table 1.

The owners of 13 (52.0%) of the dogs with secondary retrobulbar pathology reported only nasal clinical signs. There were 6 (24.0%) dogs with only oral clinical signs, and 3 (12.0%) dogs were reported to have only ocular clinical signs. There were 3 (12.0%) dogs with a combination of ocular and nasal clinical signs, and no dogs with nasal, oral, and ocular clinical signs.

On physical examination, 8 out of the 25 dogs (32.0%) with secondary retrobulbar disease were noted to have decreased ocular retropulsion and/or exophthalmos. Of these 8 dogs, the owners of 3 of them (37.5%) had not appreciated ocular involvement prior to presentation.

A primary nasal tumor was the most common diagnosis (*n* = 15/21, 71.4%) for dogs with neoplasia that secondarily invaded into the retrobulbar space. The other sites of neoplastic origin were the maxilla (*n* = 3, 14.3%), the mandible (*n* = 2, 9.5%), and the zygoma (*n* = 1, 4.8%). The nasal tumors were diagnosed as follows: carcinoma (*n* = 9), adenocarcinoma (*n* = 2), osteosarcoma (*n* = 2), fibrosarcoma (*n* = 1), and plasmacytoma (*n* = 1). The maxillary tumors were an osteosarcoma, a poorly differentiated sarcoma, and a melanoma. The 2 mandibular tumors were both malignant melanoma (Figure 5). The zygomatic arch tumor was an osteosarcoma.

One dog with a sterile abscess secondary to nasal neoplasia was treated with antibiotics but was lost to follow-up, and the other was euthanized. The dog with a maxillary cyst was treated surgically with cyst enucleation; the cyst persisted and the dog was euthanized later due to unrelated causes. The dog with retrobulbar cellulitis and abscessation secondary to maxillary molar teeth periapical infection was cured with dental extractions and antibiotics. Radiation and/or chemotherapy was pursued in 11 dogs, including 1 dog that also underwent ventral rhinotomy with bilateral nasal stent placement. The medical records reflect that 11 dogs were euthanized, 3 died at home, 1 was considered cured, and 10 were lost to long-term follow-up.

### CT findings

**Figure 6:**
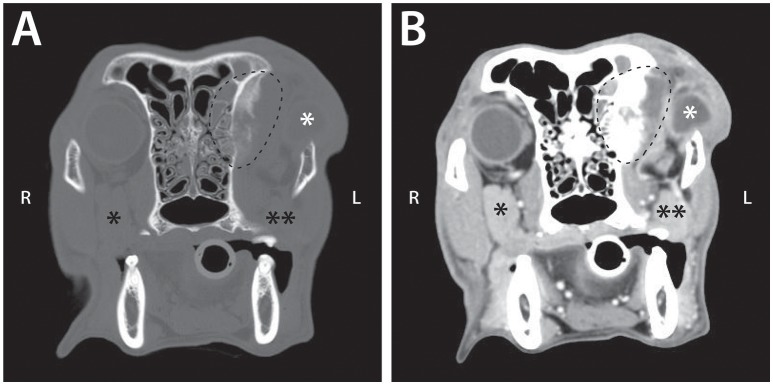
Pre **(A)** and post **(B)** intravenous contrast administration transverse CT images of a 9-year-old female spayed Chesapeake Bay retriever that was diagnosed with left retrobulbar osteosarcoma with extension into the left frontal sinus. These images demonstrate CT signs statistically significantly associated with neoplastic retrobulbar disease: osteolysis and periosteal reaction of the orbit (seen within the dashed line) and the presence of a mass (dashed line). The scan also shows exophthalmos with a misshapen globe (white ^*^), as well as compression and displacement of the zygomatic salivary gland (^**^ vs. the unaffected side [^*^]).

The frequencies of the major CT findings are summarized by disease category in Table 3. The superficial cervical lymph nodes were included in the CT scan for 7 dogs diagnosed with neoplasia and were enlarged ipsilateral to the lesion in 1 dog. The following CT findings met the criteria for analysis by Pearson's Chi-squared test for independence: orbital osteolysis, orbital periosteal reaction, zygomatic salivary gland enlargement, zygomatic salivary gland compression/displacement, retrobulbar mass effect, retrobulbar mass, exophthalmos, misshapen globe, mandibular lymph node enlargement, and medial retropharyngeal lymph node enlargement (Figure 6). Because only 4 dogs had retrobulbar abnormalities associated with trauma/other disorders, CT findings were only compared between infectious/inflammatory disease and neoplasia.

**Table 3:** Computed tomographic (CT) findings associated with retrobulbar disease in 66 dogs.

**CT finding**	**Number affected: unilateral, bilateral**		
	**Infectious/Inflammatory (*n* = 22)**	**Neoplasia (*n* = 40)**	**Trauma / Other (*n* = 4)**
Orbit: osseous distortion	0	9	3, 1
Orbit: osteolysis	2	**30**	0
Orbit: periosteal reaction	1	**18**	0
Zygomatic arch: osseous distortion	0	3	1
Zygomatic arch: osteolysis	0	3	0
Zygomatic arch: periosteal reaction	0	3	0
Zygomatic salivary gland enlargement^*^	**10, 2**	3	1
Zygomatic salivary gland compression / displacement^*^	4	16	0
Reduction of retrobulbar fat	14, 1	34	1
Retrobulbar mass effect	**14**	1	2
Retrobulbar mass	8, 1	**39**	1
Exophthalmos^†^	17, 2	27	3, 1
Enophthalmos	0	1	0
Misshapen globe^†^	10, 1	21,1	1
Mandibular lymph node enlargement^‡^	**13, 1**	8, 1	1
Medial retropharyngeal lymph node enlargement^‡^	9, 1	8, 1	1

*The total numbers of dogs included in each category are given at the top of each column. Values in bold indicate a statistically significant difference between infectious/inflammatory and neoplastic disease*.

* *Zygomatic salivary glands could not be identified in one dog with infectious/inflammatory disease, two dogs with neoplasia, and two dogs with trauma / other disease*.

† *The globe was absent from one dog with neoplasia due to prior enucleation*.

‡ *The mandibular lymph nodes could not be identified in one dog with trauma, and the medial retropharyngeal lymph nodes were not included or could not be identified in one dog with infectious/inflammatory disease, three dogs with neoplasia, and two dogs with trauma / other disease*.

Orbital osteolysis (*p* < 0.0001), orbital periosteal reaction (*p* = 0.0009), and presence of a retrobulbar mass (*p* < 0.0001) were significantly associated with neoplasia, while zygomatic salivary gland enlargement (*p* < 0.0001), retrobulbar mass effect (*p* < 0.0001), and mandibular lymphadenopathy (*p* = 0.0013) were more often associated with infectious/inflammatory disease. Compression or displacement of the zygomatic salivary gland, reduction of the retrobulbar fat, exophthalmos, misshapen globe, or medial retropharyngeal lymph node enlargement did not significantly differ between infectious/inflammatory or neoplastic disease. Osseous distortion was more commonly associated with neoplasia (9/40) than infectious/inflammatory disease (0/22), but the relationship did not achieve statistical significance (*p* = 0.0207).

Estimates of sensitivity and specificity are summarized in Table 4. The presence of osteolysis and periosteal reaction affecting the orbit were moderately sensitive for detection of retrobulbar neoplasia with few false positive observations. A distinct retrobulbar mass was highly sensitive for neoplasia but associated with frequent false positive findings. Zygomatic salivary gland enlargement and retrobulbar mass effect were moderately sensitive for diagnosis of infectious / inflammatory disease with relatively high specificity. Presence of mandibular lymph node enlargement had only moderate sensitivity and specificity for diagnosis of infectious/inflammatory disease.

**Table 4:** Sensitivity and specificity of individual CT findings for diagnosis of infectious/inflammatory disease or neoplasia.

**CT finding**	**Infectious/Inflammatory**		**Neoplasia**	
	**Sensitivity**	**Specificity**	**Sensitivity**	**Specificity**
Orbit: osteolysis	–	–	75% (60–90%)	92% (81–103%)
Orbit: periosteal reaction	–	–	45% (22–68%)	96% (88–104%)
Zygomatic salivary gland enlargement	57% (29–85%)	90% (80–100%)	–	–
Retrobulbar mass effect	64% (38–89%)	93% (85–100%)	–	–
Retrobulbar mass	–	–	98% (93–102%)	58% (32–84%)
Mandibular lymph node enlargement	64% (38–89%)	77% (63–91%)	–	–

*Values in parentheses represent the 95% confidence intervals*.

Five masses diagnosed as osteosarcoma had median pre-contrast HU of 45.0 (range: 31.3–148.7) and one infiltrative lipoma had pre-contrast HU = −94.9. Excluding these 6 tumors, HU of masses associated with infectious/inflammatory disease (median *HU* = 30.0; range = 19.2–43.1) overlapped with HU of neoplastic masses (median *HU* = 41.0; range = 23.9–113.4), although the tendency for neoplasia to have greater HU was statistically significant (*p* = 0.0076). Contrast enhancement caused similar increases in HU (*p* = 0.988) for inflammatory masses (median = 79% increase; range = 2–262%) and neoplasia (median = 103%; range = 1–266%), but patterns of contrast enhancement significantly differed between inflammatory and neoplastic masses (*p* < 0.0001). Contrast enhancement was peripheral in 9 inflammatory masses, heterogeneous in three, and no contrast enhancement was subjectively observed in one inflammatory mass. Neoplastic masses exhibited a heterogeneous pattern of contrast enhancement in 30 cases, homogeneous contrast enhancement in 1 case, and peripheral or no contrast enhancement in 1 case each.

## Discussion

The present retrospective study describes a large and diverse caseload of retrobulbar disorders in dogs. Results of the present study underscore that disorders of the retrobulbar space are varied and complex, with implications that are likely to extend beyond the orbit. In addition, diseases affecting the retrobulbar space carry vastly different prognoses and yet can cause similar clinical signs and physical examination findings. CT findings are essential to support a diagnosis, but biopsy, cytology, culture and sensitivity, and/or exploratory surgery are necessary to obtain a definitive diagnosis, which is requisite to best direct treatment and inform prognosis.

Due to varied clinical signs and physical examination findings, it may be difficult to determine that a retrobulbar disorder exists based on history and physical exam findings alone. Decreased ocular retropulsion is often thought to be pathognomonic for retrobulbar disease (5, 21, 22). However, of the 66 dogs diagnosed with primary and secondary retrobulbar disorders, 22 (33.3%) lacked diminished ocular retropulsion on physical examination (retropulsion results were either not reported, or found to be within normal limits). Thus, retrobulbar pathology of one-third of these dogs may be considered an incidental finding. This also underscores the importance of performing ocular retropulsion on all patients, and not just those presenting with a history of ocular changes.

**Figure 7:**
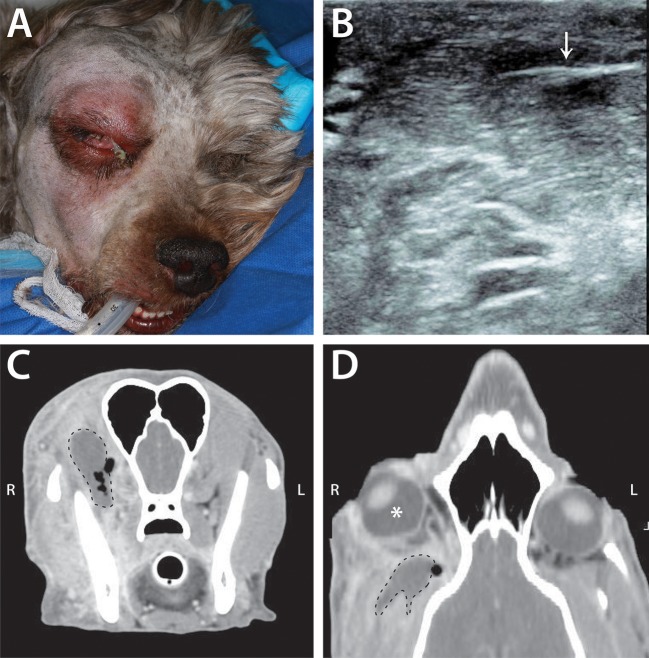
Example of infectious/inflammatory primary retrobulbar disease secondary to migrating grass awn foreign body in a 4-year-old male intact poodle mix. **(A)** Clinical appearance of the dog just prior to surgical intervention. Note the right exophthalmos and periocular swelling, chemosis, and mucoid ocular discharge. **(B)** Ultrasound image from this dog demonstrating the presence of a foreign body (arrow), consistent with plant material. **(C)** Transverse CT image of this dog, demonstrating a non-contrast enhancing mass (dashed line) with surrounding pockets of gas (black areas within and adjacent to the dashed line). **(D)** CT scan reconstructed in a dorsal plane demonstrating right exophthalmos (white *) with the retrobulbar mass (dashed line) and adjacent gas pocket.

Due to the difficulty in directly examining the retrobulbar space, advanced imaging is particularly helpful in confirming the presence of a retrobulbar lesion and determining its extent. Ultrasonography can provide information on the character of the retrobulbar lesion, guide fine needle aspiration for definitive diagnosis, and may be particularly useful for identifying foreign material (Figure 7) (31). In reviewing the 66 cases presented here, CT was shown to be particularly useful in determining lesion invasiveness and its effect on various retrobulbar structures. The information that CT yields is crucial to the veterinarian in determining additional diagnostic testing to recommend, as well as to the owner in considering their dog's potential prognosis and quality of life. Furthermore, the presence of certain CT findings may help refine the likely differential diagnoses. Specifically, the presence of orbital osteolysis or periosteal reaction indicate a greater probability of neoplasia than other diseases, although osseous changes were only present in 45–75% of patients with retrobulbar neoplasia. Importantly, 39/40 patients with retrobulbar neoplasia had a discrete mass, but this was also a common finding among other categories of disease. Conversely, displacement of retrobulbar structures without an obvious mass was more likely to be associated with infectious/inflammatory disease. It is important to consider CT as a diagnostic tool that guides additional, more definitive diagnostic testing and informs general prognosis, but not a means of ascertaining an exact etiology.

While obtaining a definitive diagnosis of retrobulbar disorders is of ultimate importance in guiding treatment and determining prognosis, the complex anatomy and proximity to vital structures makes retrieving a diagnostic sample challenging. Previously published studies have investigated the conclusiveness of diagnostic testing of the retrobulbar space. In one such study, only 20 of 34 dogs (58.8%) with confirmed retrobulbar abscesses had positive bacterial culture results (20). In another study (32), 17 of 35 (49%) fine-needle aspiration attempts from 29 dogs were diagnostic, whereas 9 of 16 (56%) biopsies were diagnostic in dogs with confirmed orbital neoplasia. In combining cytology and histopathology results from that study, a definitive diagnosis could be established in 79% of dogs. In the present study, cytology and histopathology results derived from the same lesion were not always in agreement, suggesting that it may be prudent, when clinically appropriate, to obtain both cytologic and histopathologic samples to increase the chances of securing a definitive diagnosis. A reason for non-diagnostic bacteriology, cytology, and histopathology results may be the difficulty in accessing and sampling retrobulbar tissues, some of which do not exfoliate readily. Furthermore, bacteriology results may not be positive in cases of sterile retrobulbar abscesses or inflammation. Ultrasound or CT-guidance can help with direct sampling, but even with the assistance of diagnostic imaging, there remains risk associated with sampling procedures, including hematoma formation, damage to the optic nerve (33), and equivocal results.

Out of the 66 dogs included in this study, 40 (60.6%) were diagnosed with retrobulbar neoplasia. An almost equal number of cases were primary neoplasia vs. secondary (19 vs. 21, respectively). These results are consistent with a previous study, which reported 12 primary retrobulbar neoplasms and 14 secondary (5). A different study in dogs (32), however, reported that 82% of cases (*n* = 36) were primary. Spread from the nasal cavity was the most common cause of secondary retrobulbar neoplasia in the present study. Tumors of nasal origin with secondary involvement of the orbit have been documented previously. In one study (14), 9 of 15 dogs diagnosed with retrobulbar carcinoma had tumors originating in the nasal cavity. Another study (34) reported that of 19 dogs with nasal neoplasia, retrobulbar involvement was present in 3 (15%). These findings highlight the importance of a thorough diagnostic work-up, including advanced diagnostic imaging, to assess the extent of cranial neoplasia, which may guide therapeutic recommendations and inform prognosis.

It is preferred to determine etiology of retrobulbar disease via definitive cytology, biopsy, and/or culture and sensitivity results; however, due to the retrospective nature of this study, exploratory surgery, positive response to antibiotics or anti-inflammatory medications, and/or necropsy were utilized as means to categorize retrobulbar pathology. This poses a certain limitation to this study as an argument could therefore be made that a definitive diagnosis was truly never reached for some of these cases. The retrobulbar space is inherently challenging to sample. For that reason, it is possible that fine needle aspirate or needle biopsy results may not reflect the primary disease process, especially when secondary infection or inflammation is present alongside a primary neoplasm. Similarly, some patients with neoplasia and secondary infection or inflammation could have been incorrectly included among dogs with infectious/inflammatory disease following short-term improvement with medical management.

An additional limitation of the present study is that the dogs included were evaluated at a tertiary referral center and CT examination was a criterion for inclusion; thus, these cases may not reflect retrobulbar disorders affecting the general population of dogs. The diagnosis and treatment of these dogs may have been especially challenging, as those with relatively easy to diagnose and manage retrobulbar disorders may not have warranted referral to a specialist. For example, dental disease affecting the caudal maxillary premolar and molar teeth is often implicated in retrobulbar cellulitis or abscessation and is described in the literature as being relatively commonplace (18, 20, 35); however, only 1 of the 66 dogs in this study had that etiology. It may be that this cause of retrobulbar disease is identified and effectively treated by primary veterinarians such that the cases at our institution do not reflect the true incidence of this condition. Furthermore, patients in our hospital with certain clinical signs (e.g., blindness or neurologic deficits) or due to clinician preference are more likely to undergo magnetic resonance imaging (MRI) or ocular ultrasound than CT.

In conclusion, the retrobulbar space is anatomically complex and disorders of this region can cause considerable morbidity and mortality. Disorders of the retrobulbar space are challenging to diagnose and to treat; therefore, ideally they would be managed with a team approach, as the anatomy involved and the myriad disease etiologies fall under the purview of a wide array of veterinary specialists. Finally, CT has been confirmed to be an integral component in the diagnosis and characterization of the extent of retrobulbar disease in dogs.

## Ethics statement

The study is retrospective in nature and included clinical cases, hence, it is exempt from IACUC requirements.

## Author contributions

JW and DC: Study concept and design, data acquisition, analysis, and interpretation, drafting of the manuscript, Final approval of the version to be published; FV: Study concept and design, critical revision for important intellectual content. Final approval of the version to be published; CL: Data acquisition, analysis, critical revision for important intellectual content; NV: Data acquisition, analysis, and interpretation, drafting of the manuscript, Final approval of the version to be published; KG: Data acquisition, analysis, and interpretation, drafting of the manuscript, critical revision for important intellectual content, final approval of the version to be published; CG: Drafting of manuscript, critical revision for important intellectual content. Final approval of the version to be published; BA: Study concept and design, data acquisition, analysis, and interpretation, critical revision for important intellectual content, final approval of the version to be published. Study supervision.

### Conflict of interest statement

The authors declare that the research was conducted in the absence of any commercial or financial relationships that could be construed as a potential conflict of interest.
